# “Just” Speaking Up Requires Increasing Psychological Safety

**DOI:** 10.1097/pq9.0000000000000834

**Published:** 2025-08-29

**Authors:** Brady Silva, Jared Henricksen, Gitte Larsen, Karen Talbot, Charisse Stewart

**Affiliations:** From the * Intermountain Primary Children’s Hospital, Salt Lake City, Utah; † Department of Pediatrics, University of Utah, Salt Lake City, Utah.

## Introduction:

Team psychological safety is a shared belief by team members that it is acceptable to explore possibilities, express ideas and concerns openly, speak up with questions, and share mistakes, without fear of negative consequences. Psychological safety fosters work environments that enhance team performance, innovation, creativity, resilience, and learning.

From 2020 to 2022, we observed hospital-wide decreases in safety culture engagement and event reporting. We aimed to enhance staff competency in using error prevention tools through targeted education at onboarding for employees and leaders, emphasizing foundational psychological safety principles.

## Methods:

In early 2023, our patient safety leadership team integrated the 4 stages of psychological safety—inclusion, learner, contributor, and challenger safety^1^—into staff and physician safety training and new employee orientation. This training encompasses a structured definition of each stage of psychological safety, the provision of illustrative examples, the incorporation of reflective questions, and the facilitation of robust and vulnerable discussions regarding participants’ experiences with the presence or absence of psychological safety in their work environments.

We taught caregivers to achieve challenger safety, recognizing that this takes time and leader support to navigate through the stages. Training included teaching leaders about their responsibility to model and to teach key behaviors to promote a psychologically safe culture. One year after initiation of the revised comprehensive training program, we sought insight into the outcomes of training, specifically the integration of psychological safety behaviors by caregivers.

We selected 1 medical nursing unit as a pilot unit for data collection and developed a qualitative once-a-quarter survey to assess team perceptions and levels of psychological safety. Participants were presented with 2 open-ended prompts as part of the survey: (1) “How do you personally define psychological safety?” (refer to Table 1), and (2) “Please describe a recent safety-related event or experience that involved psychological safety” (refer to Table 2).

**Table 1.** Personal Definition of Psychological Safety

Role	How Do You Personally Define Psychological Safety?
Nurse	Creating a safe environment where staff feel comfortable to ask a question and speak up
Patient care technician	Feeling comfortable and safe and unjudged by actions
Nurse	Workplace safety for all staff, not just nursing. Giving all who work at PCH to feel safe, appreciated, and valued
Patient care technician	Feeling safe to speak up, feeling included, knowing you can learn on the job/from mistakes
Nurse	Being confident in speaking up about concerns or questions I have without fear of being looked down upon
Patient care technician	Can get help from others without judgement. Feel supported by those around me
Nurse	Psychological Safety is being in a safe place to speak your mind/bring up questions that you have without fear of persecution. Being comfortable questioning anyone, no matter their role
Patient care technician	It means feeling safe speaking up about patient-safety and my own safety and needs and knowing I will be taken seriously and receive appropriate responses
Patient care technician	My mental health is not put in danger

PCH, Primary Children's Hospital.

**Table 2.** Safety Event or Experience Involving Psychological Safety

Theme	Please Describe a Recent Safety-related Event or Experience that Involved Psychological Safety
Reporting safety concerns	“When I was on a 1:1, the grandma was verbally assaulting staff. The RN, didn’t hesitate to get the teams and security involved.” “Physician having pushback with calling BERT. I continued to say how important it was before it escalated more- patient escalated during BERT”
Comfort in speaking up	“I spoke up for my patient with a chest tube that I was concerned about, the team responded and reacted appropriately which allowed me to feel comfortable raising other concerns or questions.” “I was feeling very stressed when I admitted a patient from the PICU that was very complex and needed all of my time/attention, when I finally spoke up and asked for help, I was able to feel more comfortable in the tasks that I needed to get done. I want to be a safe person for others to turn to when they need help.” “ I feel like this last year I was able to feel more confident speaking up and advocating for my patients.”
Team support and collaboration	“I was working with a new tech. I became frustrated with them. So, I purposely showed them how to chart I/O’s. In the end, it was a positive experience.” “One moment I had in the last few months was asking a provider about a patient who was experiencing delirium. I asked further questions because it was the most severe form of delirium I had seen. The Provider took time to sit with me and talk about it and explained that since the patient had lost their vision, that escalated their delirium. This impacted me and helped me learn. I can ask providers about diagnoses more often which helps everyone learning.
Mental health and well-being	“I’ve been having problems with my mental health lately and my managers, colleagues, and HR have been working with me very well to help me navigate this new obstacle.” “I feel as though this last year has been hard on my mental health while being here. I try to be as transparent in the moment as possible.”
Advocacy for patients	“One of my patients was getting the wrong feeds, I felt comfortable enough to bring up to the nurse and get it fixed.” “I had a seizure patient that didn’t have a rescue med ordered. When questioning the resident, I was told it was not necessary because ‘they’ve never needed one’. Fortunately, I pushed harder and talked with the senior who agreed it was absolutely needed for patient safety.”

## Results:

Since starting the revised safety training in November 2023, more than 90% of new staff (n = 2000) have been trained in person. Additionally, the revised safety program has been included in the ongoing training of all department leaders. Within the pilot unit, nursing staff and patient care technicians (n = 102) reported an increased awareness of both the presence and absence of psychological safety across multidisciplinary teams.

We identified significant self-reported improvements in 4 domains. These included (1) reduced anxiety and fear of failure; (2) overall job satisfaction and better relationships with colleagues; (3) enhanced innovation and collaboration among team members; and (4) improved staff retention.

## Conclusions:

Findings suggest that the integration of psychological safety principles into new hire safety training has positively influenced workplace culture, fostering a more engaged, innovative, and resilient workforce. This initiative is still in its early phases, with limitations in available quantitative data. Further research is necessary to assess long-term effects of foundational psychological safety training on patient safety outcomes.
